# EHMTI-0221. Tension type headaches and chronic pain syndrome

**DOI:** 10.1186/1129-2377-15-S1-C62

**Published:** 2014-09-18

**Authors:** HR Vekilyan, AA Karapetyan, AH Karapetyan, EM Gevorgyan, HM Manvelyan

**Affiliations:** 1 Neurology, Yerevan State Medical University, Yerevan, Armenia; 2 Family Medecine, Yerevan State Medical University, Yerevan, Armenia

## Introduction

Tension type headaches (TTH) are the most common forms of primary headaches. The prevalence of TTH is about 80 % among cephalgic pain.

## Aims

The aim of study was investigation of intercoupling of TTH and chronic pain syndrome (CPS).

## Methods

Method Questionnaires on screening of headaches (according to recommendations of International Headache Society) were blindly distributed among 3000 people in the country. 603 (458 women/ 145men) patients were selected for the study with primary headaches (migraine, tension type and cluster headache). Age of participants was 42±16 years.

## Results

Data analysis revealed that 387 patients (64,2 %) (107 males and 280 females) suffer of TTH, 204 migraine (33,8%) (36 males and 168 females), 12 (2%) (8 males and 4 females) cluster headache. The coincidence with CPS was revealed in 232 patients with TTH (60%) and in 40 patients with migraine (20%).

## Conclusions

Our data reveal non significant difference of distribution of primary headaches in Armenia, but it shows that CPS is present in more cases of chronic TTH and/or combination of TTH with other primary headaches. Further investigation on simultan CPS and TTH must be continued, as both could have common mechanisms and development, as well treatment options.

No conflict of interest.

